# Preventing malaria in the Peruvian Amazon: a qualitative study in Iquitos, Peru

**DOI:** 10.1186/s12936-018-2177-9

**Published:** 2018-01-16

**Authors:** Ian Newell, Connie Wiskin, James Anthoney, Graciela Meza, Gilles de Wildt

**Affiliations:** 1 College of Medical and Dental Sciences, University of Birmingham, Birmingham, UK; 2 Institute of Clinical Sciences, University of Birmingham, Birmingham, UK; 3 Facultad de Medicina Humana, Universidad Nacional de la Amazonia Peruana, Iquitos, Peru

**Keywords:** Malaria, Prevention, Qualitative, Bed nets, Indoor residual spraying, Space spraying, Peru

## Abstract

**Background:**

In Peru, despite decades of concerted control efforts, malaria remains a significant public health burden. Peru has recently exhibited a dramatic rise in malaria incidence, impeding South America’s progress towards malaria elimination. The Amazon basin, in particular the Loreto region of Peru, has been identified as a target for the implementation of intensified control strategies, aiming for elimination. No research has addressed why vector control strategies in Loreto have had limited impact in the past, despite vector control elsewhere being highly effective in reducing malaria transmission. This study employed qualitative methods to explore factors limiting the success of vector control strategies in the region.

**Methods:**

Twenty semi-structured interviews were conducted among adults attending a primary care centre in Iquitos, Peru, together with 3 interviews with key informants (health care professionals). The interviews focussed on how local knowledge, together with social and cultural attitudes, determined the use of vector control methods.

**Results:**

Five themes emerged. (a) Participants believed malaria to be embedded within their culture, and commonly blamed this for a lack of regard for prevention. (b) They perceived a shift in mosquito biting times to early evening, rendering night-time use of bed nets less effective. (c) Poor preventive practices were compounded by a consensus that malaria prevention was the government’s responsibility, and that this reduced motivation for personal prevention. (d) Participants confused the purpose of space-spraying. (e) Participants’ responses also exposed persisting misconceptions, mainly concerning the cause of malaria and best practices for its prevention.

**Conclusion:**

To eliminate malaria from the Americas, region-specific strategies need to be developed that take into account the local social and cultural contexts. In Loreto, further research is needed to explore the potential shift in biting behaviour of *Anopheles darlingi*, and how this interacts with the population’s social behaviours and current use of preventive measures. Attitudes concerning personal responsibility for malaria prevention and long-standing misconceptions as to the cause of malaria and best preventive practices also need to be addressed.

**Electronic supplementary material:**

The online version of this article (10.1186/s12936-018-2177-9) contains supplementary material, which is available to authorized users.

## Background

Malaria remains an important public health problem in the Americas, with an estimated 132 million people at risk of infection, and 21 million at high risk [1]. In South America, 70% of reported malaria cases are caused by *Plasmodium vivax*, which commonly has lower mortality rates than *Plasmodium falciparum*, and thus has been neglected globally despite causing large-scale morbidity [2]. However, *P. falciparum* remains an important cause of morbidity and mortality [3]. The Amazon basin is estimated to have 44% of malaria cases in the Americas [4]. A large proportion of Peru falls within the Amazon basin and thus Peru has one of the highest incidences of malaria in South America [1]. In 2016, the Pan American Health Organization (PAHO) announced a plan of action for eliminating malaria in the Americas, which specifically targets the Amazon basin [3]. This plan of action is based on the World Health Organization (WHO) Global Technical Strategy for Malaria 2016–2030 [5], including the two core broadly-applicable vector control interventions of long-lasting insecticidal nets and, where appropriate, indoor residual spraying. Peru’s large burden of malaria is, therefore, the main impediment to progress towards malaria elimination in South America.

This significant malaria problem in Peru occurs despite decades of concerted control efforts [6]. Previously, Peru succeeded in achieving a high level of malaria control and on two occasions reduced the number of cases to such an extent that elimination appeared feasible [7]. However, Peru experienced a near 100% increase in annual malaria incidence from 2010 to 2015 [8] with reported incidences of *P. vivax* and *P. falciparum* of 111 and 41 per 100,000 inhabitants and 34,992 and 12,999 cases, respectively, in 2016 [9], which was greater than almost all malaria-endemic nations in the Americas [1, 10]. This suggests that the Peruvian national strategy is failing [5], and as a result, Peru still remains in the control stage of malaria elimination [11]. 94% of the national burden of malaria occurs in the region of Loreto [12], located in the Amazon basin, where annual malaria case numbers increased significantly from 2010 to 2014 [13], suggesting that the focus of Peru’s malaria control strategy should be in Loreto. A key component of this strategy must be to identify why established control methods in Loreto, based on the WHO three pillars of malaria (test, treat, track [14]) have had limited success to date.

The basic principles for controlling *P. vivax* malaria are the same as those for *P. falciparum* malaria [15]—vector control, chemoprevention, and prompt diagnosis and treatment—although many of the interventions are not as effective for *P. vivax*, due to differences in life cycle and vector biting behaviour in non-African settings. Vector control, the most effective mechanism for malaria prevention and control [16–18] is critical since the *P. vivax* gametocytes often appear before a patient develops symptoms and seeks treatment, so that transmission can continue despite the best efforts at prompt diagnosis and treatment of cases. The two most commonly deployed vector control methods are insecticide treated nets (ITNs) and indoor residual spraying (IRS) [1], with both methods employed by the Ministry of Health in Peru [1, 19]. In Loreto, research into vector control has focussed primarily on mosquito behaviour [20, 21], concluding that *Anopheles darlingi*, the main vector for malaria there, was increasingly biting outdoors. As a result, IRS is not generally used for malaria prevention there [15, 22, 23]. One of the studies also identified an increase in infected mosquitoes biting in the early evening [21]. This change in mosquito behaviour could influence the effectiveness of ITNs and other control measures. However, no research has assessed the interaction between changing biting times, behaviours of those at risk, and current vector control strategies. Research addressing people’s understanding of when mosquitoes bite, how to prevent them biting and how both relate to common human behaviours is, therefore, crucial. The only qualitative research conducted in Loreto on malaria vector control that was found compared preference between traditional and long-lasting insecticide-treated bed nets (LLINs) [24]. While the study identified that bed net preference can limit the optimal use of LLINs, it did not address how local knowledge, together with social and cultural attitudes, determines the use of vector control methods.

Although there has been considerable research addressing factors which influence malaria vector control uptake in sub-Saharan Africa [25], no such research was found that has been carried out in Peru. Peru is currently developing strategies to eliminate malaria in the Amazon basin, comprising (1) universal access to malaria prevention interventions, vector management and malaria diagnosis and treatment; (2) reinforced malaria surveillance; (3) strengthened health systems; (4) strategic advocacy, communications and collaborations; and (5) focused efforts to facilitate malaria elimination and prevent re-establishment in malaria-free areas [5]. Before finalizing and implementing such strategies, additional research is essential to understand the appropriateness of current and planned vector control interventions in the region [5], acknowledging that control and elimination strategies depend on population acceptance and coverage [26].

This qualitative study, therefore, aims to explore community attitudes towards current malaria preventive measures to aid the targeted delivery of interventions to address population-specific needs in the Loreto region. The objectives of the study were to: (1) understand participants’ knowledge of mosquito behaviour and control strategies; (2) elicit attitudes towards bed nets and other methods of malaria prevention; (3) understand how knowledge of and attitudes towards malaria vector control strategies interact with social behaviour to influence their effectiveness.

## Methods

### Setting

The study was conducted in the San Juan district of Iquitos, a district with both urban and rural areas in the Loreto region of Amazonian Peru. Malaria prevalence exhibits significant heterogeneity across Loreto, where *P. vivax* and *P. falciparum* infections have been reported to occur at a ratio of 4:1 [27], and where the reported incidences of *P. vivax* and *P. falciparum* were 3193 and 1236 cases per 100,000 inhabitants, corresponding to 33,507 and 12,977 cases, respectively, in 2016 [9]. San Juan has the highest number of reported malaria cases in the region [28], where the Peruvian Ministry of Health has responsibility for malaria control.

### Study design

Qualitative, semi-structured interviews were used to facilitate in-depth exploration of knowledge, attitudes and subsequent behaviours focussed on malaria prevention by allowing participants to freely express their opinions uninhibited by the opinions of others [29]. Key informant interviews were also conducted with malaria health care professionals (HCPs) to triangulate participants’ responses.

### Sampling and population

Interviews were conducted among adults attending the Centro de Salud San Juan, a primary care centre in Iquitos. Initially, convenience sampling was used, but when it became clear that due to the demographics of clinic attendees, the sample would be predominately young women, purposive sampling was used to increase numbers of men and older people to obtain maximum variation in responses from participants [30].

Participants were interviewed between January and March 2017. The number of participants interviewed was determined by the time and resource constraints of the study; and a related study suggested analytical saturation might be achieved around this number [31]—although the concept of saturation has recently been questioned [32].

Inclusion criteria for this study were: participants ≥ 18 years; and speak either Spanish or English fluently. Respondents were excluded if they were not permanently resident in the Loreto region; did not have the capacity to give informed consent; or were too unwell to participate in the interview.

In addition, interviews with nurses with joint responsibility for both treating malaria and providing malaria preventive education were used to gain first-hand information regarding malaria prevention in the population under investigation. These interviews particularly focussed on gaining insight to the specific nature of the problems expressed by participants and how they could be solved [33].

### Recruitment

Clinic HCPs were used as gatekeepers, to recruit participants based on inclusion/exclusion criteria [34], and to purposively sample participants according to easily definable variables such as gender, age, residence and occupation.

### Data collection: interviews

Interviews were conducted face-to-face in a private room using a semi-structured topic guide (see Additional file 1) that allowed respondents to address issues salient to them [35]. Questioning was preceded by simple demographic questions to understand the context from which participants came.

Interviews were conducted by IN, through a translator, in Spanish. The translator, who spoke fluent English and Spanish, was recruited from a language school in Iquitos. The translator was briefed to maintain confidentiality in accordance with a signed agreement. A single translator was used throughout to ensure consistency. The participant information sheet, consent form and topic guide were written in English and translated into Spanish prior to data collection; the topic guide was then reviewed by the local translator for clarity and comprehensibility.

The topic guide was developed to investigate a broad range of issues relating to malaria knowledge, attitudes, and preventive practices. It was piloted in the first day of interviewing, to determine the clarity and acceptability of questions [36]. Passages of the audio-recording and transcription from the pilot interview were used to check the accuracy of translation. The topic guide was edited to address any problems, especially interview misdirection [30], identified during the pilot, and to allow exploration of emerging themes. Interviews lasted between 20 min and an hour, limiting the demands on the participant but ensuring adequate information was obtained. All interviews were audio-recorded, subject to participants’ written agreement. Following each interview, IN recorded any reflections and field notes relating to the case to enhance the trustworthiness and accountability of the research [37].

### Transcription and data analysis

All English components of the interview recordings were transcribed by IN on the same day as the interview. An independent check of translation accuracy was made for 10% of all transcripts.

To allow a flexible approach to analysis, the analytic approach employed was thematic content analysis, carried out by IN. To develop and refine the themes, the ‘6-step’ inductive analysis structure outlined by Braun and Clarke was used [38]:

1. Familiarisation with and immersion in the data.
2. Generation of initial codes from patterns in the data.
3. Collation of all generated codes into associated themes.
4. Reviewing of themes to ensure mutual exclusivity and that themes relate to the codes and data set.
5. Defining and refining themes by analysing the data contained within each theme.
6. Writing up the final analyses for publication.

Data collection and analysis occurred concurrently, with analysis an iterative process to assess saturation and inform the sampling for subsequent interviews.

The difficulties in re-contacting participants and the short duration of the study precluded respondent validation [39]. To check the credibility of findings, emerging themes were discussed with local HCPs, who were culturally aligned with the participants. To achieve analytical triangulation, an additional researcher (JA) coded a sample of transcripts to independently develop codes and compare them with emerging themes. Codes were compared and any discrepancies reconciled. JA had access only to anonymized data.

## Results

Around 40% of people approached informally agreed to participate, the remainder generally citing insufficient time. Following detailed explanation of the study, all such participants agreed to be involved, and consented to the interview being audio recorded. The independent check of translation accuracy found no substantial discrepancies. Twenty interviews were conducted, but no new themes emerged after the 14th interview. In addition, three key informants were interviewed.

### Demographic characteristics

Participants comprised 10 men and 10 women, all permanently resident in Iquitos, with the majority living in San Juan. Participants were recruited to have a range of age, education and occupation (Table 1). Participants’ ages ranged from 18 to 80 years. Despite studies identifying that, in Loreto, malaria disproportionately affects adult men [5], no gender-specific findings were identified.

**Table 1:** Participant demographics

Variables	N = 20 (%)
Gender	
Male	10 (50)
Female	10 (50)
Age	
18–24	6 (30)
25–34	6 (30)
35–44	2 (10)
45–54	3 (15)
55–64	1 (5)
65–74	1 (5)
≥ 75	1 (5)
Education	
None completed	0 (0)
Primary	5 (25)
Secondary	5 (25)
Tertiary	10 (50)
Occupation	
Housewife	5 (25)
Student	5 (25)
Businessman	4 (20)
Fisherman	1 (5)
Other employed	3 (15)
Unemployed	2 (10)

### Themes

Five themes were derived from the coding process and are presented below. In descriptors following quotes, KI indicates Key Informant, and P indicates other (non-KI) participant.

#### a. A culture of malaria

A majority of participants emphasized that, in Iquitos, it is normal to have malaria. People commonly accepted that being infected with malaria was inevitable, in particular because they felt mosquitoes would always remain in their city:

*I think it is the nature of this place, it is the jungle so mosquitoes are always here*

[P20]

*People cannot avoid the mosquito biting*

[P13]

Key informants emphasized the local population’s perception of the ordinariness of malaria within their society:

*The people here [in Iquitos] think malaria is a very normal disease. People commonly have malaria 5 times in their life if not more.*

[KI1]

*Everyone is born and grows up with malaria here*

[KI3]

The other participants also saw malaria as a habitual disease within their society, and this attitude was commonly associated with a lack of regard for malaria prevention:

*I think they [people in Iquitos] don’t do anything to prevent malaria because it is easier for them. Maybe because the disease is always here.*

[P2]

This participant suggested people made little effort to prevent malaria because they felt the disease would never be eliminated. The ongoing presence of malaria, irrespective of use of preventive measures, was also stressed by other participants:

*I think this illness will never end here. In the past there was a lack of knowledge about this illness but even now this disease is increasing more and more.*

[P14]

Participants seemed resigned to the fact that malaria remained embedded within their society and people made little effort to prevent it because they believed infection was inevitable. Participants were keen to emphasize that until this attitude was addressed, people saw no hope of eliminating mosquitoes, and hence malaria, from the region:

*Education is very important and is the responsibility of the government to provide people with information. But you have to start by changing attitudes because perhaps people already have the information but don’t do anything with it. Maybe the attitude needs to change.*

[P19]

In addition, participants reported that the symptoms of malaria had become unremarkable. One participant suggested that people no longer identified the potential severity of the disease, and commonly did not acknowledge the symptoms:

*Some of the people who I work with had malaria and died due to this illness. They had shivers and headaches but they did nothing to stop it because they thought it was normal to feel this way. But in fact they had malaria and for that reason they died.*

[P6]

Malaria appears to have become normalized within the region and people no longer saw it as an important health condition. Although most participants believed the normalisation of malaria had negative impacts on personal protective behaviour, three participants indicated that, because malaria has always been so common, the use of bed nets had become part of their culture:

*[I use bed nets] because it is a habit, it is part of my cultural background, my mother and my grandmother passed this habit on to me.*

[P12]

*In Iquitos it is very common that people use bed nets because it is part of my culture especially in the rural zones*

[P13]

*I need them [bed nets] to sleep, it is what I have always done as part of my background*

[P16]

These participants indicate that there is consistent uptake of bed nets amongst families who have traditionally used them.

#### b. Biting protection dissonance

Mosquitoes do not bite 24 h a day, but have preferred biting times during which protection is essential [40]. Formerly, *An. darlingi’s* habitual biting time peaked at 10 pm in the region [16], when local people were generally asleep under bed nets. However, both participants and key informants believed this biting time might now be occurring earlier:

*there are certain times for high transmission. The highest is considered between 5 and 9 in the evening.*

[KI1]

In the evenings, during these reported earlier peak biting times, people are generally indoors, but unprotected by bed nets.

*And do people use bed nets all the time?* IN *[Only] at night for sleeping*

[P15]

*At this time I am usually watching the TV but I am not using my bed net.*

[P11]

*Yes it is a problem [that I am not protected at these times] because when I take dinner it is very difficult to eat because I am being bitten by mosquitoes.*

[P6]

Observation of local infrastructure indicated that doors and windows of houses were not commonly screened. Many participants expressed concerns that, despite the majority of mosquito bites occurring in the early evening, they were protected by their nets only at night. They acknowledged this to be a serious problem but could think of no solution with bed nets only suitable for use at night:

IN:
*Are you usually using your bed nets at this time [between 5pm and 9pm]?*
P12:
*No*
IN:
*Is this a problem?*
P12:
*Yes*
IN:
*And why don’t you use your bed nets at this time?*
P12:
*Because when I watch TV I am in the main room but the bed nets are in the bedroom.*

Participants were also engaged in other activities during the reported peak biting times:

*One day in the past I went to the river to go for a swim and that was the moment I got malaria. At this time the fever, shivers and pain all started.*

[P13]

This was highlighted as a serious concern by one key informant:

*All the people when I visit families in their house do not have much time to listen and they don’t pay attention or practice what I teach. Some people commonly take a shower in the river at 7 o’clock and this is a bad practice but they don’t listen.*

[KI1]

#### c. Malaria prevention as the government’s responsibility

Participants were asked who they felt was responsible for their health, and in particular for preventing malaria. A majority believed the government should be responsible for maintaining their health, both in general and specifically for malaria prevention:

*A person on their own cannot do anything, it is the responsibility of the government on providing information to prevent this illness [malaria].*

[P16]

A participant who had only recently moved to Iquitos expressed his frustration that, in Iquitos specifically, people did not hold their health of high importance. He felt that this attitude was inhibiting malaria control:

*People here don’t care about their health, people should focus more on their health…maybe because I have lived in Lima which is a very different context compared to Iquitos. Really I think it is the attitude that people have here though. Here people do not have the attitude to fight this illness [malaria] face to face they just don’t really care.*

[P16]

A majority of participants confirmed that, with regard to malaria prevention in particular, they held little personal responsibility:

*The government should do more to prevent malaria. They should provide more information, they should teach us about the treatment and use the community nurses more. We need more information about prevention, the symptoms and medications to treat it.*

[P14]

*People here are very dependent on the government but the government or the community does nothing.*

[P16]

This lack of regard for personal prevention was acknowledged as serious problem by one HCP:

*People don’t think it is their role to prevent this illness.*

[KI1]

However, the view that the government should be held entirely responsible for malaria prevention was not held by all. A few participants, all of whom had completed tertiary education, recognised malaria prevention as a personal responsibility:

*I think every person should take control of their own health.*

[P18]

*I think it [responsibility for malaria prevention] depends on the level of education that people have because those who know about malaria take action themselves. But there are still people that know malaria is a dangerous illness but still they don’t do anything to prevent it. I think education is very important, so the information provided to the family about malaria is important.*

[P2]

Two participants stated that personal experience of malaria heightened their perception of risk and motivation for personal preventive measures:

*No one is doing anything to eliminate this illness, the nurses the government no one because the illness never goes away. People should try to learn about this illness, but to focus them the person needs to have this illness.*

[P19]

*For me malaria is more important than my neighbours but in the future if one of my neighbours gets malaria then they may try to understand and focus on this problem*

[P17]

Several participants believed that the government should take more responsibility for malaria prevention in rural areas, because individual prevention activities were less effective there. Several people from urban areas regarded malaria prevention in urban areas as unnecessary, even though they understood that mosquitoes were present throughout (urban) Iquitos. As a result, they commonly expressed the view that preventive measures should be focussed solely in the rural areas:

*The government should also focus on the rural zones because these people are more exposed to this illness because they are surrounded by the jungle where the mosquito lives.*

[P20]

IN:
*Do you think the government should be doing more?*
[P9]:
*Yes yes yes. Especially in the rural areas. These people need more prevention and more attention from the government.*

#### d. Confusion surrounding space-spraying and IRS

Space-spraying (referred to as fumigation by participants) is used primarily for the control of dengue in this region [20, 41]; this was confirmed by a senior vector control manager. However, participants frequently referred to its use for the control of malaria:

*I think fumigation is the best way to prevent malaria*

[P9]

*The fumigation people come to my house 2 or 3 times in a year. I like fumigation because it is a good prevention of malaria.*

[P13]

This misconception regarding the purpose of fumigation in Iquitos held true across a majority of participants. Even so, there was a common belief that fumigation was effective only in the short term:

*The fumigation is only good for 20* *min then the mosquitoes appear again.*

[P15]

*I think it is a waste of time really. Because as soon as the fumigation finishes then the mosquitoes appear again.*

[P15]

In fact, one participant felt that fumigation was the only thing the government did to prevent malaria, despite there being a policy of free insecticide impregnated bed net provision and significant public education programmes:

*I think the government are doing a good job with the fumigation because they do come to my house. This is the only thing the government does to prevent malaria.*

[P13]

#### e. Persisting misconceptions

Participants commonly expressed perceptions that were not aligned with principles of malaria control. These well-established ideas had not been dispelled despite repeated education programmes over many years.

Although all participants correctly indicated that mosquitoes were one of the causes of malaria, their knowledge of how the disease was transmitted varied significantly. Ten participants believed drinking “bad water” to be a cause of malaria and it was commonly believed that cleaning the house and boiling drinking water were the best preventive measures:

*I think malaria lives in the water. For example, if we wash the chicken we are preparing for eating in contaminated water then potentially the eggs of the mosquitoes end up in our stomach. After eating we start to get the pains of malaria. So I think the best ways to prevent malaria are cleanliness and providing information about malaria.*

[P2]

*When you drink water from the river, that is how you get malaria. That is how my parents got malaria.*

[P6]

*I think one of the ways malaria is transmitted is when we drink bad water.*

[P8]

Those who attributed the cause of malaria to ‘bad water’ often did so because of the strong association in Iquitos between the seasonal rising of the river and the related increase in mosquito numbers:

*I think that the rising of the river is very important because the mosquitoes live in this standing water. When it rains then the number of mosquitoes that live in this still water can increase.*

[P7]

Participants commonly mentioned cleaning the house as one of the best preventive measures for malaria:

*The nurses that teach us about malaria give us information about taking out the rubbish and cleaning our houses, cleanliness is very important to us.*

[P20]

*Cleanliness is the best way to prevent malaria.*

[P10]

Some participants correctly incorporated the removal of mosquito breeding sites into their cleaning routine:

*They [the community nurses] tell you about emptying containers with standing water in, how to clean the house and the dishes and to take out the garbage or the rubbish. This is because most often the mosquitoes live in the standing water. I think the best way to prevent malaria is cleanliness and cleaning.*

[P2]

Thus, the concept of cleanliness as a preventive measure for malaria had clearly been understood differently by different participants. The ambiguity of the term cleanliness, aimed to teach people to remove mosquito breeding sites, was demonstrated by one of the community nurses:

*Cleanliness is very important because when we visit people’s houses we commonly find dirty houses with garbage and other rubbish. We also find standing, dirty water as well. It is important to remove this and to avoid creating a ‘house for the mosquitoes’ and we need to remove where the mosquitoes live. This is a very important reason why the nurses emphasize cleanliness to avoid still water and rubbish.*

[KI2]

## Discussion

This qualitative research on malaria prevention in Iquitos identified five key themes: a culture of malaria; biting protection dissonance; malaria prevention as the government’s responsibility; confusion surrounding space-spraying and IRS; and persisting misconceptions.

A culture of malaria is apparent in three ways. Malaria is perceived as a normal part of life; people do not perceive the symptoms of malaria as a cause for concern; and because of these perceptions, people do not go to sufficient lengths to protect themselves. This corresponds with findings from sub-Saharan Africa that malaria remains a socially normalized disease [42], but this concept has rarely been reported in South America. This view that malaria is a mild, treatable illness has major implications for progression towards malaria elimination in the region. While symptoms continue to be considered a routine part of normal life, in accordance with other everyday illnesses, there will be no social pressure to prevent malaria and the population’s focus will remain on treatment rather than prevention.

As part of the normalisation of malaria within this society, the use of bed nets for sleeping at night has become part of local culture, passed down from one generation to the next; and respondents with this tradition reported being unable to sleep without a bed net. Despite this culture, the perceived risk of malaria appears to be low. Studies elsewhere have identified the perception of risk as a strong determinant for net use [43]. It will be important during plans for elimination to ensure that understanding of the risks of malaria are increased, and reinforcing current attitudes to bed net use to ensure people remain protected during sleep. It will also be helpful to strengthen and broaden this cultural use of bed nets amongst the sub-groups of the local population who are not already using them, including immigrants to the region.

Biting protection dissonance seems to be becoming an important issue in malaria control in the region. While traditionally the peak period for biting was 10p.m. and bed nets provided effective protection during this period since this was when people normally slept [16], respondents reported that now the peak biting period has moved earlier, to 5p.m.–9p.m., when people are commonly not protected. This concurs with recent findings from Haiti [44], where it has been suggested that widespread bed net use at night naturally selected for earlier-biting mosquitoes, and with a recent study in the peri-Iquitos region that alludes to increased early-evening and outdoor-biting of *An. darlingi* [16]. This shift in peak biting times may undermine the effectiveness of bed nets, and a need for alternative preventive interventions. A possible alternative is to encourage increased use of window and door screens, although construction of housing in the region may preclude this without first blocking open eaves [45], which has been demonstrated to be protective against some malaria vectors [46]. It is important to emphasize that using bed nets for sleeping remains essential, since mosquitoes may continue to bite at night and peak biting times could easily revert to night-time. Initially, there is a need for research to confirm whether biting times have shifted or whether this is a local misperception before addressing the dissonance between times of protection and times of peak biting.

Participants almost unanimously regarded malaria prevention as the government’s responsibility, diminishing personal responsibility for malaria control. They also expressed their belief in a need for greater government intervention in rural areas where malaria incidence is higher. Previous studies suggest that societies that emphasize governmental responsibility for health may take low personal responsibility for protective measures and have increased passivity in terms of control [47], thereby burdening society with the cost of treating malaria. While promoting personal responsibility for general health is a well-developed approach, there is limited little research investigating its effectiveness for malaria prevention, other than a study identifying that most malaria preventive practices are personal measures (diligent bed net use, protective clothing), and that taking personal responsibility for malaria prevention empowers people to protect themselves [48]. The demand by participants for educational interventions targeted towards the most disadvantaged areas aligns with significant evidence suggesting education can increase personal responsibility for malaria prevention [49], with the probability of dying from malaria inversely related to education and income [50].

Confusion surrounding fumigation was a recurrent theme, with participants commonly mistaking space-spraying (which they call fumigation), used in this region for the prevention of dengue, with IRS for the prevention of malaria. However, space-spraying has a limited effect on the most prevalent malaria vector in the region, *An. darlingi*, which generally rests and bites outside [16]. While there is no evidence that this confusion is affecting people’s mosquito-avoidance behaviour, it is clear that some people believe themselves to be protected from malaria due to fumigation and, therefore, do not practice personal protection measures. Furthermore, respondents also reported that the effect of fumigation on mosquito infestation was very short-term and that mosquitoes remained in unsprayed areas. This perception is consistent with findings from qualitative reports on IRS [51]. There is a risk that a loss of confidence in fumigation, because of perceived ineffectiveness against malaria, may lead to a reduction in fumigation adherence and thus reduced protection against dengue.

Despite Peruvian malaria control programmes dating back to the early twentieth century [4], several persisting misconceptions held true across many participants, influencing the potential effectiveness of control strategies and suggesting that educational interventions might need redirection. Participants still attributed malaria to ‘bad water’ or contaminated food, which is a historical misconception [52, 53], suggesting that, as elsewhere, years of educational intervention have not been fully successful in changing knowledge or behaviours. Furthermore, participants commonly identified cleaning the house as one of the best preventive measures for malaria, identified as an incorrect perception in The Roll Back Malaria Behaviour Change Indicator [54]. Clearly these misunderstandings have not yet been fully rectified. It is largely agreed that attributing malaria to several causes, many of which are not easily influenced, makes people more unwilling to participate in control activities [47], and this may contribute significantly to the general belief that malaria is an inevitable illness within this Amazonian society. While improved knowledge is not generally sufficient to change behaviour, it is usually necessary: refocussed education is therefore essential to ensure these misconceptions are rectified if the effectiveness of elimination strategies is to be maximized. However, it should be appreciated that behaviour change is multifactorial, complex and difficult, so it is not surprising that these misunderstandings continue to persist.

Addressing these issues will require substantial behaviour change interventions to challenge and change deep held beliefs concerning malaria in the region. Substantial implementation research is needed to develop interventions appropriate for the region, building on the existing motivation for change that this study has demonstrated.

## Limitations

This study has two main limitations. First, interviews were conducted using a translator. Qualitative research aims to find meaning in experiences but as a result of language differences, understanding and interpretation of those meanings may have been affected [55]. One translator was used throughout to ensure consistency. Translation accuracy checks performed in the pilot and following the completion of all interviews found no evidence of problems caused by translation.

Second, participants tended to generalize about other members of their community. This may have been due to participants perceiving the interviewer as an ‘outsider’ from a different culture, and using the interview to introduce the interviewer to their culture [56]. The effect of this is unclear; on one hand, reported behaviour of others might have been inaccurate; on the other, people might have talked more freely about other people’s behaviour than about their own, allowing exploration of cultural norms, a technique often used in vignettes [57].

## Conclusion

This study helps to inform those responsible for researching and addressing the complex issue of malaria control in Peru. Improved understanding of region-specific issues, and identification of culturally-appropriate strategies to address them, are likely to increase the effectiveness of local malaria control. Further research is needed to explore the shift in biting behaviour of *An. darlingi* and how this aligns with protective behaviour. Strategies also need to be developed to change attitudes concerning personal responsibility for malaria prevention and change misconceptions about the causes of malaria and hence the best preventive practices. Such strategies should be adaptable to reflect the local social and cultural determinants of malaria. Although this study was conducted in Loreto, the findings are likely to be relevant to other areas of the Amazon. Without addressing the problems identified in this research, malaria elimination is unlikely to be achieved in the Americas.

## Additional file

**Additional file 1.** Summary of interview topic guide.

## References

[1] WHO. World malaria report 2016. Geneva: World Health Organization; 2016. http://www.who.int/malaria/publications/world-malaria-report-2016/report/en/. Accessed 30 Mar 2017.

[2] Baird JK (2007). Neglect of *Plasmodium vivax* malaria. Trends Parasitol.

[3] Rosas-Aguirre A, Speybroeck N, Llanos-Cuentas A, Rosanas-Urgell A, Carrasco-Escobar G (2015). Hotspots of malaria transmission in the Peruvian Amazon: rapid assessment through a parasitological and serological survey. PLoS ONE.

[4] Pan American Health Organization (PAHO). Strategy and plan of action on malaria. 55th Directing Council of PAHO, 68th session of the regional committee for the Americas. 2016. http://www.paho.org/hq/index.php?option=com_content&view=article&id=12276%3A2016-55th-directing-council-documents&catid=88113Adc-documents&Itemid=42078&lang=en. Accessed 10 Nov 2016.

[5] WHO. Global technical strategy for malaria 2016–2030. Geneva: World Health Organization; 2015. http://apps.who.int/iris/bitstream/10665/176712/1/9789241564991_eng.pdf?ua=1&ua=1. Accessed 10 Nov 2016.

[6] Griffing SM, Gamboa D, Udhayakumar V (2013). The history of 20th century malaria control in Peru. Malar J..

[7] Quispe AM, Llanos-Cuentas A, Rodriguez H, Clendenes M, Cabezas C, Leon LM (2016). Accelerating to zero: strategies to eliminate malaria in the Peruvian Amazon. Am J Trop Med Hyg.

[8] Ministerio de Salud del Perú (2015). Tendencia y situación de las enfermedades sujetas a vigilancia epidemiológica: malaria. Bol Epidemiol..

[9] Ministerio de Salud del Perú (2016). Tendencia y situación de las enfermedades sujetas a vigilancia epidemiológica: malaria. Bol Epidemiol..

[10] Rosas-Aguirre A, Gamboa D, Manrique P, Conn J, Moreno M (2016). Epidemiology of *Plasmodium vivax* in Peru. Am J Trop Med Hyg.

[11] Krisher LK, Krisher J, Mariano A, Arichabala A, Beltrán-Ayala E, Navarrette P (2016). Successful malaria elimination in the Ecuador–Peru border region: epidemiology and lessons learned. Malar J..

[12] Ministerio de Salud del Perú-Dirección General de Epidemiología (MINSA-DGE) (2015). Situational room report: epidemiological week 31.

[13] Swiss Tropical and Public Health Institute. Report from community and country level consultations on GMAP2 ‘Action and Investment to defeat Malaria (AIM)’ in Peru. 2014. http://www.rollbackmalaria.org/files/files/aim/consultive_process/Peru_%20Report.pdf. Accessed 17 Oct 2016.

[14] WHO. T3: Test. Treat. Track initiative. Geneva: World Health Organization; 2012. http://www.who.int/malaria/areas/test_treat_track/en/. Accessed 16 Apr 2017.

[15] WHO. Control and elimination of *Plasmodium vivax* malaria—a technical brief. Geneva: World Health Organization; 2015. http://www.who.int/malaria/publications/atoz/9789241509244/en/. Accessed 10 Apr 2017.

[16] Lengeler C (2004). Insecticide-treated bed nets and curtains for preventing malaria. Cochrane Database Syst Rev..

[17] Pluess B, Tanser FC, Lengeler C, Sharp BL (2010). Indoor residual spraying for preventing malaria. Cochrane Database Syst Rev..

[18] Roll Back Malaria (RBM). Multisectorial action framework. 2013. http://www.rollbackmalaria.org/about-malaria/multisectoral-action-framework/action-framework. Accessed 9 Nov 2016.

[19] Direccion General de Salud de las Personas (2015). Norma tecnica de salud para la atencion de la malaria y malaria grave en el Peru.

[20] Anticona Huaynate CF, Pajuelo Travezaño MJ, Correa M, Mayta Malpartida H, Oberhelman R, Murphy LL (2015). Diagnostics barriers and innovations in rural areas: insights from junior medical doctors on the frontlines of rural care in Peru. BMC Health Serv Res..

[21] Moreno M, Saavedra MP, Bickersmith SA, Lainhart W, Tong C, Alava F (2015). Implications for changes in *Anopheles darlingi* biting behaviour in three communities in the peri-Iquitos region of Amazonian Peru. Malar J..

[22] Dzul-Manzanilla F, Ibarra-López J, Marin WB, Martini-Jaimes A, Leyva JT, Correa-Morales F (2017). Indoor resting behavior of *Aedes aegypti* (Diptera: Culicidae) in Acapulco, Mexico. J Med Entomol..

[23] United States Agency for International Development. Peru vector control needs assessment report. 2012. http://pdf.usaid.gov/pdf_docs/PA00J21Q.pdf. Accessed 16 Apr 2017.

[24] Grietens KP, Muela Ribera J, Soto V, Tenorio A, Hoibak S, Aguirre AR (2013). Traditional nets interfere with the uptake of long-lasting insecticidal nets in the Peruvian Amazon: the relevance of net preference for achieving high coverage and use. PLoS ONE.

[25] Maslove D, Mnyusiwalla A, Mills E, McGowan J, Attaran A, Wilson K (2009). Barriers to the effective treatment and prevention of malaria in Africa: a systematic review of qualitative studies. BMC Int Health Hum Rights.

[26] Atkinson JM, Fitzgerald L, Toaliu H, Taleo G, Tynan A (2010). Community participation for malaria elimination in Tafea Province, Vanuatu: part I. Maintaining motivation for prevention practices in the context of disappearing disease. Malar J..

[27] Rosas-Aguirre A, Gamboa D, Manrique P, Conn JE, Moreno M, Lescano AG (2016). Epidemiology of *Plasmodium vivax* malaria in Peru. Am J Trop Med Hyg.

[28] Oficina General de Epidemiologia, DIRESA Loreto. Análisis de la situación de salud de la region Loreto año 2010. 2010. http://www.dge.gob.pe/portal/Asis/indreg/asis_loreto.pdf. Accessed 9 Nov 2016.

[29] Kvale S (1996). Interviews.

[30] Coyne IT (1997). Sampling in qualitative research. Purposeful and theoretical sampling; merging or clear boundaries?. J Adv Nurs.

[31] Frank AL, Beales ER, de Wildt G, Sanchez GM, Jones LL (2017). “We need people to collaborate together against this disease”: a qualitative exploration of perceptions of dengue fever control in caregivers of children under 5 years, in the Peruvian Amazon. PLoS Negl Trop Dis..

[32] Emmel N (2013). Sampling and choosing cases in qualitative research.

[33] Tremblay MA (1957). The key informant technique: a non-ethnographic application. Am Anthropol..

[34] Seidman I (2013). Interviewing as qualitative research.

[35] Holloway I (2005). Qualitative research in health care.

[36] Weiss RS (1994). Learning from strangers: the art and method of qualitative interviewing.

[37] Finlay L (2002). Negotiating the swamp: the opportunity and challenge of reflexivity in research practice. Qual Res.

[38] Braun V, Clarke V (2013). Successful qualitative research.

[39] Mays N, Pope C (2000). Qualitative research in health care: assessing quality in qualitative research. BMJ.

[40] WHO. Vector control: methods for use by individuals and communities. Geneva: World Health Organization; 1997. http://www.who.int/water_sanitation_health/resources/vector007to28.pdf. Accessed 16 Apr 2017.

[41] WHO. Dengue control: control strategies. Geneva: World Health Organization. http://www.who.int/denguecontrol/control_strategies/chemical_control/en/. Accessed 16 Apr 2017.

[42] Jones CO, Williams HA (2004). The social burden of malaria: what are we measuring?. Am J Trop Med Hyg.

[43] Koenker HM, Loll D, Rweyemamu D, Ali AS (2013). A good night’s sleep and the habit of net use: perceptions of risk and reasons for bed net use in Bukoba and Zanzibar. Malar J..

[44] Steinhardt LC, St Jean Y, Impoinvil D, Mace KE, Wiegand R, Huber CS (2017). Effectiveness of insecticide-treated bednets in malaria prevention in Haiti: a case–control study. Lancet Glob Health..

[45] Paredea-Esquivel C, Lenhart A, del Rio R, Leza MM, Estrugo M, Chalco E (2016). The impact of indoor residual spraying of deltamethrin on dengue vector populations in the Peruvian Amazon. Acta Trop.

[46] Njie M, Dilger E, Lindsay SW, Kirby MJ (2009). Importance of eaves to house entry by anopheline, but not culicine, mosquitoes. J Med Ent..

[47] Resnik DB (2007). Responsibility for health: personal, social, and environmental. J Med Ethics.

[48] Antwi GD, Bates LA, King R, Mahama PR, Tagbor H, Cairns M (2016). Facilitators and barriers to uptake of an extended seasonal malaria chemoprevention programme in Ghana: a qualitative study of caregivers and community health workers. PLoS ONE.

[49] Roll Back Malaria (RBM). Education and malaria. 2015. http://www.rollbackmalaria.org/about-malaria/sustainable-development-goals/malaria-sdgs-fact-sheets. Accessed 11 Apr 2017.

[50] Tusting LS, Willey B, Lucas H, Thompson J, Kafy HT, Smith R (2013). Socioeconomic development as an intervention against malaria: a systematic review and meta-analysis. Lancet.

[51] Munguambe K, Pool R, Montgomery C, Bavo C, Nhacolo A, Fiosse L (2011). What drives community adherence to indoor residual spraying (IRS) against malaria in Manhiça district, rural Mozambique: a qualitative study. Malar J..

[52] Mitchell VS, Pearson GW, Carpenter CCJ (1991). Malaria: obstacles and opportunities.

[53] Cox F (2010). History of the discovery of the malaria parasites and their vectors. Parasites Vectors..

[54] Roll Back Malaria (RBM). Behaviour change communication indicator reference guide. 2014. http://archiverbm.rollbackmalaria.org/toolbox/tool_MalariaBCCIndicatorsGuide.html. Accessed 11 Apr 2017.

[55] van Nes F, Abma T, Jonsson H, Deeg D (2010). Language differences in qualitative research: is meaning lost in translation?. Eur J Ageing.

[56] Mullings B (1999). Insider or outsider, both or neither: some dilemmas of interviewing in a cross-cultural setting. Geoforum.

[57] Finch J (1987). The vignette technique in survey research. Sociology.

[58] Health Research Authority. Qualitative protocol guidance and template. 2015. http://www.hra.nhs.uk/about-the-hra/consultations-calls/closed-consultations/qualitative-protocol-guidance-and-template/. Accessed 10 Nov 2016.

