# Supplementary material for: Preventing malaria in the Peruvian Amazon: a qualitative study in Iquitos, Peru
Source: Malar J. 2018 Jan 16;17:31. doi: 10.1186/s12936-018-2177-9 (PMC5771103; doi:10.1186/s12936-018-2177-9)
Supplement: Supplementary file 1 — Additional file 1. Summary of interview topic guide. [file 12936_2018_2177_MOESM1_ESM.docx]

**Additional file 1: Summary of Interview Topic Guide**

1. **Malaria: knowledge and attitudes**
   1. Have you ever heard of malaria?
   2. What do you know about malaria?
   3. What do you think about malaria? Why do you think this?
   4. Do other people think this about malaria?
2. **Malaria Prevention**
   1. How do you get malaria?
   2. What are the ways to prevent malaria?
   3. What do you feel about these ways of preventing malaria?
   4. Which of these methods do you use?
   5. Why do you use these preventive measures?
   6. Why don’t you use other preventive measures?
   7. When do mosquitos usually bite?
   8. Where are you when mosquitos usually bite?
   9. What are you usually doing when you get bitten by mosquitos?
   10. Does where you are and what you’re doing influence why you use certain preventive measures?
   11. Are there other factors which influence why you use certain measures?
   12. Whose responsibility is malaria prevention?
